# The Konno-Rastan Procedure in the Current Era: Still A Feasible Option?

**DOI:** 10.21470/1678-9741-2024-0002

**Published:** 2025-02-05

**Authors:** Safak Alpat, Timucin Sabuncu, Ahmet Aydin, Murat Guvener, Riza Dogan, Mustafa Yilmaz

**Affiliations:** 1 Department of Cardiovascular Surgery, Division of Pediatric Cardiac Surgery, Hacettepe University School of Medicine, Ankara, Turkey; 2 Department of Cardiovascular Surgery, Hacettepe University School of Medicine, Ankara, Turkey

**Keywords:** Mitral Valve, Left Ventricular Outflow Obstruction, Pathologic Constriction, Hear Valve Diseases, Subvalvular Aortic Stenosis, Hypertrophic Cardiomyopathy, Aorta

## Abstract

**Introduction:**

The Konno-Rastan procedure is a commonly used surgical technique to address complicated subaortic stenosis and to enlarge the aorta via an anterior route. The objective of this report is to describe the experience of our unit with this procedure.

**Methods:**

Between 2002 and 2022, we identified pediatric patients who underwent the Konno-Rastan procedure. Relevant information was retrospectively collected.

**Results:**

There were 16 patients who underwent the Konno-Rastan procedure. The median follow-up was nine years (4 - 16 years), and there was no operative mortality. All patients had a straightforward recovery, and five patients required reoperation at follow-up, none of which was related to the left ventricular outflow tract. The median echocardiographic indices at the most recent follow-up were not significantly different from the preoperative values, with a median peak gradient across the left ventricular outflow tract of 25 mmHg. In their most recent follow-up, 81% of the patients were New York Heart Association class 1. No bleeding, thromboembolic, or infective complications were encountered.

**Conclusion:**

We concluded that the Konno-Rastan procedure can be regarded as a definitive alternative to other surgical interventions for left ventricular outflow tract obstructions. Although reoperations are still necessary, particularly in patients with additional mitral valve disease, the Konno-Rastan procedure is still a viable option in patients with complex left ventricular disease.

## INTRODUCTION

Complex left ventricular outflow tract obstruction (LVOTO) is characterized by the presence of varying degrees of obstruction within the outflow tract, which may occur in the subvalvular, valvular, or supravalvular parts. The surgical management of individuals with obstruction poses significant challenges, often necessitating multiple reoperations to alleviate the condition^[1,2,3]^.

The Konno-Rastan procedure is a commonly used surgical technique to treat complicated subaortic stenosis and to expand the aortic root through an anterior route. Aortic ventricular infundibular plasty, often known as the Konno technique, was first introduced by Konno in 1975 and later detailed by Rastan in 1976^[4,5]^. It was initially conceived as a viable option for managing tunneltype subaortic stenoses. Subsequently, it became the treatment of choice for treating multilayer LVOTO, particularly in cases with a small aortic annulus and those necessitating reoperation^[6,7,8,9,10]^. Using a pulmonary valve autograft (Ross-Konno operation) rather than a mechanical valve as in the Konno-Rastan procedure in children has several advantages. However, multiple reinterventions for graft failure in both the aortic and pulmonary valve positions are possible with the Ross-Konno procedure^[11,12,13,14]^. The current report describes our unit's long-term experience with the Konno-Rastan procedure.

## METHODS

### Cohort

From 2002 to 2022, 16 pediatric patients underwent the Konno-Rastan procedure. Informed consent was obtained, and the institutional review board approved the study (SBA 24/790). We retrospectively collected relevant demographic data, disease characteristics, preoperative echocardiographic and/ or angiographic variables, operational data, and postoperative factors, such as vasoactive inotrope scores, rhythm traces, time to extubation, and length of stay in critical care and in hospital from patients. Follow-up was complete in all patients. Echocardiographic parameters, international normalized ratio (INR) values, and reoperations were identified during the follow-up. In terms of echocardiographic variables, transthoracic echocardiography was used to assess ventricular and valvular function using standard views as recommended by the American Society of Echocardiography^[15,16]^.

### Surgical Technique

Having performed the median sternotomy, cardiopulmonary bypass (CPB) is instituted. Under cardioplegic arrest, a longitudinal aortotomy incision is made in the aortic annulus well to the left of the right coronary artery, which divides the commissure between the right and left coronary cusps in cases with normal coronary ostial origin. Between the right ventricle and aorta, a transverse incision is made on the free wall of the right ventricular outflow tract (RVOT) beneath the pulmonary valve. To relieve subaortic stenosis, the aortotomy is extended into the interventricular septum between the annulus of the pulmonary valve and the medial papillary muscle. This incision preferentially stays above the medial papillary muscle to preserve tricuspid valve function and prevent heart block. Furthermore, the incision was not too close to the pulmonary valve to prevent leaflet injury or dysfunction due to deformation. The aortic valve is then completely removed. A portion of Dacron® or bovine pericardium is sutured to the ventricular septum using mattress sutures reinforced with felt strips on the right ventricular side or continuous sutures, based on the surgeon's preference. The enlarged annulus of the aortic valve is implanted with an appropriate size SJM Regent™ series aortic valve mechanical prosthesis using horizontal mattress sutures on the exterior of the patch. The longitudinal aortic incision is covered by the superior part of the aortoventriculoplasty patch, and a second patch is used to close the RVOT defect^[17,18,19]^.

Statistical analysis was performed using Jamovi Version 2.3.18.0 (Jamovi Projects) software. For continuous data, descriptive statistics are presented as median and interquartile range.

## RESULTS

Sixteen patients underwent the Konno-Rastan procedure. Thirteen of them were male (81%) and three were female (19%). At the time of Konno-Rastan, the median age and weight were 10 years (3 - 17 years) and 27 kg (21.5 - 44 kg), respectively. Table 1 summarizes the demographic and clinical characteristics of the patients. The main diagnoses for surgery were complex LVOTO with subaortic stenosis in eight patients and valvular aortic stenosis and/or regurgitation with a small aortic annulus in the other half. Sixteen patients underwent 24 previous operations or percutaneous interventions in total. The details of these operations are shown in Table 2. The aortic valve morphology was bicuspid in nine patients. The preoperative maximum gradient in the left ventricular outflow tract (LVOT) was 100 mmHg (97.5 - 130 mmHg) with an aortic annulus size *z*-score of -3 (-3.625 - -2.375). Most of the patients (62.5%) had moderate aortic valvular regurgitation, whereas it was severe in four patients.

**Table 1:** Patient characteristics.

**Demographics**	
Age (median, IQR)	10 (3-17)
Sex, male (%)	13 (81)
Height (cm) (median, IQR)	132.5 (118.75-160.5)
Weight (kg) (median, IQR)	27 (21.5-44)
BSA (kg/m^2^) (median, IQR)	1.06 (0.85-1.36)
**Preoperative variables**	
Initial diagnosis (%)	
Multilevel LVOTO	8 (50)
AS, AR, annular hypoplasia	8 (50)
Morphology of the aortic valve, BAV (%)	9 (56.25)
Aortic annulus size (mm)	13 (12.75-14)
Aortic annulus *z*-score	-3 (-3.625 - -2.375)
Preoperative LVOT gradient (mmHg)	100 (97.5-130)
Degree of AR	
Mild	2
Moderate	10
Severe	4
EF (%)	75 (68-85)
FS (%)	46 (35-52)
LVEDDi	30.85 (22.44-39.16)
LVESDi	14.98 (10.15-17.64)
**Intraoperative variables**	
Bypass time (min)	146.5 (123-176.5)
Cross-clamping time (min)	129 (92.25-148.5)
Valve size (mm)	
19	6
21	6
23	1
25	3
Additional procedure	1
**Postoperative variables**	
MV time (hrs)	6 (6-8)
ICU stay (days)	2.5 (2-3.25)
In-hospital stay (days)	4.5 (4-6)
Vasoactive inotropic scores	3 (0-5)

AR=aortic regurgitation; AS=aortic stenosis; BAV=bicuspid aortic valve; BSA=body surface area; EF=ejection fraction; FS=fractional shortening; ICU=intensive care unit; IQR=interquartile range; LVEDDi=left ventricular end diastolic diameter index; LVESDi=left ventricular end systolic diameter index; LVOT=left ventricular outflow tract; LVOTO=left ventricular outflow tract obstruction; MV=mitral valve

**Table 2:** List of procedures patients have previously undergone.

Previous procedures	n
Aortic valve related	
Subaortic stenosis repair	6
Percutaneous balloon valvuloplasty	13
Surgical valvotomy/valvuloplasty	2
Aortic valve replacement	1
Mitral valve related	
Valve repair	1
Aorta related	
Repair of aortic coarctation	1

The median time interval between the last LVOT intervention and the Konno-Rastan was three years (1 - 5.5 years). In terms of the operative details, the median durations of CPB and cross-clamping were 146.5 minutes (123 - 176.5 minutes) and 129 minutes (92.25 - 148.5 minutes), respectively. In terms of implanted valve sizes, six patients received 19 mm, six patients had 21 mm, one patient had 23 mm, and the other three had 25 mm implants. Two patients also underwent concomitant mitral valve repair with Konno-Rastan procedure. For septal, LVOT, and RVOT reconstruction, Dacron® graft material was used in six patients operated before 2010, and the bovine pericardium was used in the remaining 10 patients.

In all patients, the postoperative course was uncomplicated. Nine (56%) patients required inotropic support, with a median vasoactive inotropic score of 5, and all inotropes were discontinued by the 12^th^ hour after the operation. There was no evidence of atrial or ventricular arrhythmia in the early postoperative period. We were able to extubate all patients in a median duration of six hours (6 - 8 hours), and the median length of stay in the intensive care unit (ICU) was 2.5 days (2 - 3.25 days). The hospital stay was also relatively short with a median duration of 4.5 days (4 - 6 days). Four patients (%25) experienced postpericardiotomy syndrome (PPS) within the first four weeks of operation. All these patients had a Dacron® patch for reconstruction.

**Fig. 1:**
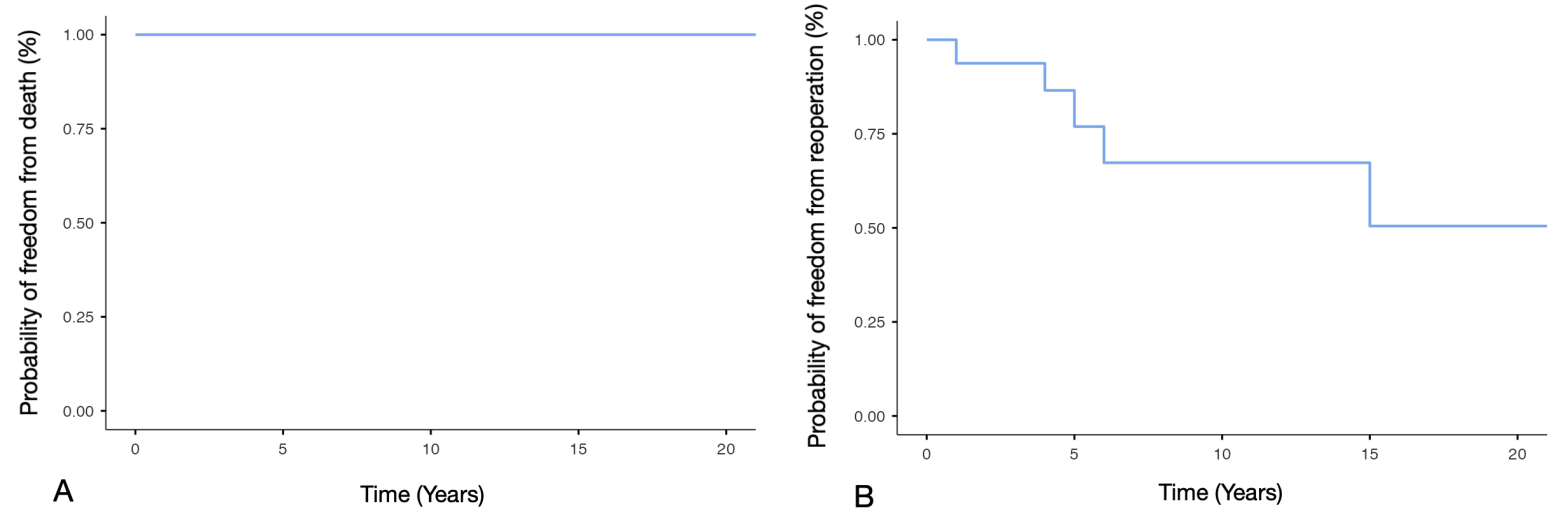
Kaplan-Meier curves for freedom from death (A) and reoperation (B).

During the median follow-up of nine years (4 - 16 years), there was no operative mortality. All patients, except one, survived without arrhythmia documented with Holter recordings. This patient required permanent pacemaker implantation in the second year postoperatively. Apart from this, there were four other reoperations in the follow-up period. Two patients underwent mitral valve replacement with mechanical prosthesis in their fourth and 15^th^ postoperative years. Both patients had previously undergone concurrent mitral valve repair with Konno-Rastan procedure. Two other patients underwent tricuspid valve replacement with mechanical prosthesis in the fifth and sixth postoperative years. These patients had moderate degree of regurgitation immediately after Konno operation, which was managed medically until they both developed severe tricuspid valve regurgitation at the time of reoperation. All these reoperations were also straightforward, and the postoperative course was uneventful. Thirteen (81%) patients were New York Heart Association (NYHA) class 1, and the remaining three (19%) were NYHA class 2 at their last follow-up. Details of these reoperations are given in Table 3. The median INR during follow-up was 2.87 (2.75 - 2.98). There were no cerebral or noncerebral embolic events, no major bleeding, and no prosthetic valve endocarditis during follow-up (Figure 1).

**Table 3:** List of reoperations during the follow-up.

Patients	Etiology	Reoperation	Postoperative years	ICU stay (days)	In-hospital stay (days)
#1	Complete heart block	Permanent pacemaker implantation	2 years	-	3
#2	Mitral regurgitation	Mitral valve repair	4 years	2	6
#3	Mitral regurgitation	Mitral valve replacement	15 years	2	5
#4	Tricuspid regurgitation	Tricuspid valve replacement	5 years	1	6
#5	Tricuspid regurgitation	Tricuspid valve replacement	6 years	1	6

**Fig. 2:**
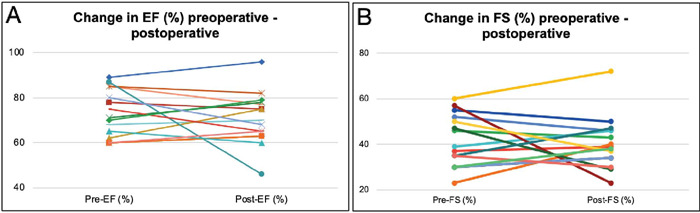
Change in ejection fraction (EF) (%) (A) and fractional shortening (FS) (%) (B) of individual patients before Konno operation and at the last follow-up.

Taking into account the echocardiographic indices, the ejection fraction and fractional shortening values at the last follow-up were 72.5% (65 - 77.75%) and 39.5% (34.75 - 46%) and those were not significantly different from the preoperative values (Figure 2). Excluding the two patients who underwent tricuspid valve replacement, 13 patients (93%) had less than severe tricuspid valve regurgitation at the most recent follow-up. All patients had less than moderate pulmonary valve regurgitation. The maximum LVOT gradient was 25 mmHg (25 - 40 mmHg).

## DISCUSSION

As the Konno-Rastan procedure has been shown to effectively relieve LVOTO in patients with diffuse subvalvular aortic stenosis, it is often viewed as the last available option after multiple unsuccessful attempts to relieve complex LVOTO with less invasive catheter-based or surgical methods. In recent years, there has been a decrease in the use of Konno-Rastan surgery, with a preference for alternative surgical procedures that are associated with less anatomical distortion, such as the Ross-Konno operation^[18,19,20]^. Ross-Konno operation requires less incision into the interventricular septum with less widening of the LVOT and is considered a preferred option when the aortic annulus is very small. Thus, the Konno-Rastan procedure remains the preferred surgical intervention for addressing LVOTO at many levels^[13,14,18,20,21]^. Although Konno-Rastan surgery has demonstrated both safety and efficacy, it is important to acknowledge the risks associated with this technique. These complications include prosthetic valvular dysfunction, infective endocarditis, heart blocks, congestive heart failure, secondary valvular problems (tricuspid and pulmonary valve regurgitations), and sudden death^[17,21,22,23,24,25]^.

During the past three decades, the mortality rate associated with the Konno-Rastan operation with mechanical valves has decreased from 25% to 7.5%. Recent articles reported that early mortality ranged from 0% to 7.4%^[24,25,26,27,28]^. In our study, we did not have postoperative or late mortality during the follow-up period. It has been previously reported that predictors of mortality include greater preoperative symptoms and longer bypass time^[24,25,28]^. One article showed that a preoperative peak LVOT gradient > 90 mmHg is associated with a risk of mortality^[24]^. Our cohort had mild symptoms before the operation and most often presented with mixed aortic valve stenosis and regurgitation. All patients had some form of previous procedure, in the form of percutaneous or surgical intervention, with six patients (37.5%) having > 2 previous interventions before Konno-Rastan. Although we had a higher preoperative peak gradient LVOT (median 100 mmHg) in our cohort, it did not worsen the outcome. Depending on the report, variations between our research results and those of other studies may be attributable to smaller sample numbers, shorter follow-up periods, or distinct study populations^[24,25,26,27,28]^. Perfusion and ischemic times were comparable to those reported in the literature. The postoperative course in our cohort was straightforward with a short stay in the ICU, intubation time, hospital stay, and low vasoactive inotropic score similar to that reported in the literature.

Four patients developed PPS within the first four weeks of operation. Note that all those patients had Dacron® patches for reconstruction. It is known that individuals who undergo aortic valve and/or thoracic aortic surgery have a stronger immune response and are more likely to develop PPS^[29,30]^. However, because all patients with PPS in our cohort had reconstruction of the ventricular septum and RVOT with Dacron® material, it could be related to the material we used. All patients with PPS were successfully treated medically and recovered well. Since then, we have been using the bovine pericardial patch for reconstruction and have not seen any PPS.

In the literature, the reintervention rate associated with LVOT procedures has been observed to vary between 0% and 17%. Furthermore, the overall reintervention rate, including various types of interventions, has been reported to range from 0% to 39.6% within the median follow-up period ranging from 1.7 to 8.5 years^[12,14,24,25,26,27,28]^. Note that the reintervention rate was higher in patients who underwent surgery to repair aortic valve disease in conjunction with annular hypoplasia^[10,12,28]^. Residual ventricular septal defect (VSD) closures, mitral valve repair or replacement, tricuspid valve repair or replacement, arch interventions, and pulmonary valve replacement are some of the other known reoperation causes reported in the literature^[10,12,24,25,26,27,28]^.

We did not have any LVOT-related reintervention within the median follow-up of nine years, although in half of our cohort, the main indication for Konno-Rastan was aortic valve disease with annular hypoplasia. We propose that two factors led to this result. First, the minimum implanted valve size was a 19-mm SJM Regent™ type mechanical prosthesis, which is known to have a better hemodynamic profile and a larger effective orifice area than usual standard mechanical valves. Second, close individual INR follow-up yielded a median INR of 2.87 during follow-up. Similarly, Erez et al.^[20]^ found no reoperation of the aortic valve.

We had five reoperations during follow-up. One patient required permanent pacemaker implantation in the second postoperative year after being incidentally diagnosed with complete heart block during the regular follow-up. This patient was reported to be discharged with sinus rhythm, and sinus rhythm was maintained until the last follow-up. The pacemaker placement rate after the Konno procedure has been reported to range between 0% and 13%^[10,12,24,25,26,27,28]^. Similar to our technique, Sakamoto et al.^[21]^ reported no atrioventricular block by performing a 5-mm proximal septal incision parallel to the pulmonary valve annulus of the medial papillary muscle. To avoid leaflet injury or dysfunction due to deformation, the incision should not be too close to the pulmonary valve, as Suri et al.^[22]^ discovered additional cases of pulmonary regurgitation in six (12%) patients following the Konno procedure, with 50% requiring pulmonary valve replacement. Erez et al.^[20]^ also reported one (7%) replacement pulmonary valve after the Konno procedure. At the most recent follow-up, none of our patients had significant pulmonary regurgitation and none required pulmonary valve surgery.

We had two mitral valve replacements four and 15 years after the Konno-Rastan procedure. Both patients underwent mitral valve repair at Konno-Rastan due to accompanying mitral valve stenosis. Two other patients underwent tricuspid valve repairs five and six years after Konno-Rastan. These two patients had aortic *z*-scores of -3.4 and -3.5. We proposed that deep ventricular septal incision and the amount of enlargement might have impaired normal functioning of the tricuspid tension apparatus, leading to tricuspid valve regurgitation commonly seen after the Konno-Rastan procedure. However, all these reoperations were straightforward, and the patients recovered well after the surgeries. Although residual VSD-related reoperations are also commonly seen during follow-up, we did not encounter this in our cohort.

### Limitations

The limitations of this study include its retrospective and observational nature in a tertiary referral center and the small number of subjects over a long period of time. As a result, patient selection and management were inconsistent. Furthermore, there was no comparison with another surgical method. Finally, the number of deaths and reoperations limited the detailed statistical analysis. Further prospective multicenter research may help overcome these limitations.

## CONCLUSION

Given the preserved left ventricular (LV) function and acceptable LVOT gradients at the most recent follow-up, we conclude that Konno-Rastan mechanical prosthesis surgery in children can be performed with acceptable mortality and morbidity in this difficult group of patients. Similarly, several recent reports concluded that LV function is preserved with acceptable LVOT gradients at the long-term follow-up after the Konno-Rastan procedure^[26,27,28]^. The Konno-Rastan procedure completely and permanently relieves LVOTO and is a viable choice because it prevents recurring LVOT surgeries. It is known that the gradient over the prosthesis in pediatric patients receiving it increases with time, inevitably leading to a reoperation later in life^[24,25,26,27,28]^. We have not encountered this occurring within a median follow-up of nine years. Therefore, thorough follow-up is required in this cohort of patients. Although reoperations are still necessary, particularly in patients with additional mitral valve disease, we strongly believe that meticulous surgical technique and adequate annulus size are of utmost importance in preventing LVOT-related reinterventions.

Although some surgeons consider the Konno-Rastan surgery to be old and obsolete, it has the ability to provide intact valve and LV function in our cohort with no significant risk of mortality or reoperation during long-term follow-up. More research is needed to determine the pathophysiologic mechanism for tricuspid valve regurgitation following Konno-Rastan surgery, the status of the mechanical valve, and the condition of the left ventricle during subsequent follow-up.

## Abbreviations, Acronyms & Symbols

AR: Aortic regurgitation
AS: Aortic stenosis
BAV: Bicuspid aortic valve
BSA: Body surface area
CPB: Cardiopulmonary bypass
EF: Ejection fraction
FS: Fractional shortening
ICU: Intensive care unit
INR: International normalized ratio
IQR: Interquartile range
LV: Left ventricular
LVEDDi: Left ventricular end diastolic diameter index
LVESDi: Left ventricular end systolic diameter index
LVOT: Left ventricular outflow tract
LVOTO: Left ventricular outflow tract obstruction
MV: Mitral valve
NYHA: New York Heart Association
PPS: Postpericardiotomy syndrome
RVOT: Right ventricular outflow tract
VSD: Ventricular septal defec
